# Supplementary material for: Isolation of Anti-Inflammatory and Epithelium Reinforcing Bacteroides and Parabacteroides Spp. from A Healthy Fecal Donor
Source: Nutrients. 2020 Mar 27;12(4):935. doi: 10.3390/nu12040935 (PMC7230855; doi:10.3390/nu12040935)
Supplement: Supplementary file 1 [file nutrients-12-00935-s001.pdf]

**Table S1**. Bacterial isolates and their codes used in this study. Data from the whole genome sequencing (WGS) using MiSeq.

| Isolate | Code | Genome size (Mb) | GC content (%) | Number of contigs | Number of contigs >500 |
|---|---|---|---|---|---|
| *Parabacteroides distasonis 1* | Pd1 | 5.0 | 45.1 | 98 | 65 |
| *Parabacteroides distasonis 2* | Pd2 | 5.0 | 45.1 | 89 | 65 |
| *Parabacteroides distasonis 3* | Pd3 | 5.0 | 45.1 | 142 | 107 |
| *Parabacteroides distasonis 4* | Pd4 | 5.0 | 45.1 | 120 | 94 |
| *Parabacteroides distasonis 5* | Pd5 | 5.0 | 45.1 | 223 | 197 |
| Parabacteroides distasonis 6 | Pd6 | 5.0 | 45.1 | 812 | 721 |
| *Bacteroides caccae 1* | Bc1 | 5.2 | 42.7 | 88 | 69 |
| *Bacteroides caccae 2* | Bc2 | 5.2 | 42.7 | 83 | 64 |
| Bacteroides caccae 3 | Bc3 | 5.2 | 42.7 | 81 | 69 |
| *Bacteroides fragilis* | Bf | 5.4 | 43.4 | 126 | 58 |
| *Bacteroides intestinalis 1* | Bi1 | 6.0 | 42.7 | 94 | 78 |
| *Bacteroides intestinalis 2* | Bi2 | 6.0 | 42.6 | 4138 | 3005 |
| *Bacteroides intestinalis 3* | Bi3 | 6.0 | 42.7 | 88 | 77 |
| *Bacteroides uniformis* | Bu | 4.7 | 46.5 | 154 | 130 |
| *Bacteroides vulgatus 1* | Bv1 | 5.2 | 42.3 | 335 | 310 |
| *Bacteroides vulgatus 2* | Bv2 | 4.4 | 43.4 | 2057 | 1813 |
| *Bacteroides vulgatus 3* | Bv3 | 5.2 | 42.3 | 138 | 113 |
| *Bacteroides vulgatus 4* | Bv4 | 5.2 | 42.3 | 159 | 112 |
| Bacteroides vulgatus 5 | Bv5 | 5.2 | 42.3 | 216 | 119 |
| *Bacteroides ovatus 1* | Bo1 | 6.9 | 41.9 | 506 | 98 |
| *Bacteroides ovatus 2* | Bo2 | 6.9 | 42.0 | 286 | 91 |
| *Bacteroides ovatus 3* | Bo3 | 6.9 | 41.9 | 241 | 98 |
| *Bacteroides ovatus 4* | Bo4 | 6.9 | 41.9 | 212 | 139 |
| *Bacteroides ovatus 5* | Bo5 | 6.9 | 41.9 | 186 | 98 |
| *Bacteroides ovatus 6* | Bo6 | 6.9 | 42.0 | 157 | 97 |
| *Bacteroides ovatus 7* | Bo7 | 6.9 | 42.0 | 466 | 292 |
| *Bacteroides ovatus 8* | Bo8 | 6.9 | 42.0 | 196 | 102 |
| *Bacteroides ovatus 9* | Bo9 | 6.9 | 41.9 | 317 | 95 |

A.

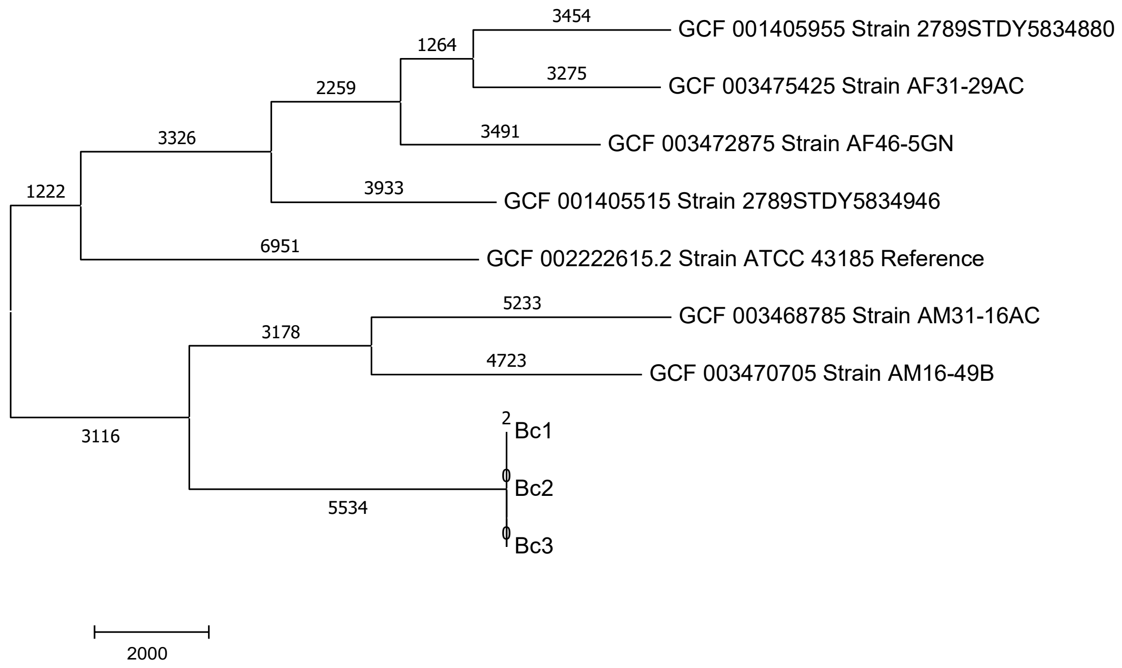
3454
GCF 001405955 Strain 2789STDY5834880
1264
3275
GCF 003475425 Strain AF31-29AC
2259
3491
GCF 003472875 Strain AF46-5GN
3326
3933
GCF 001405515 Strain 2789STDY5834946
1222
6951
GCF 002222615.2 Strain ATCC 43185 Reference
5233
GCF 003468785 Strain AM31-16AC
3178
4723
GCF 003470705 Strain AM16-49B
3116
2
Bc1
0
Bc2
5534
0
Bc3
2000

B.

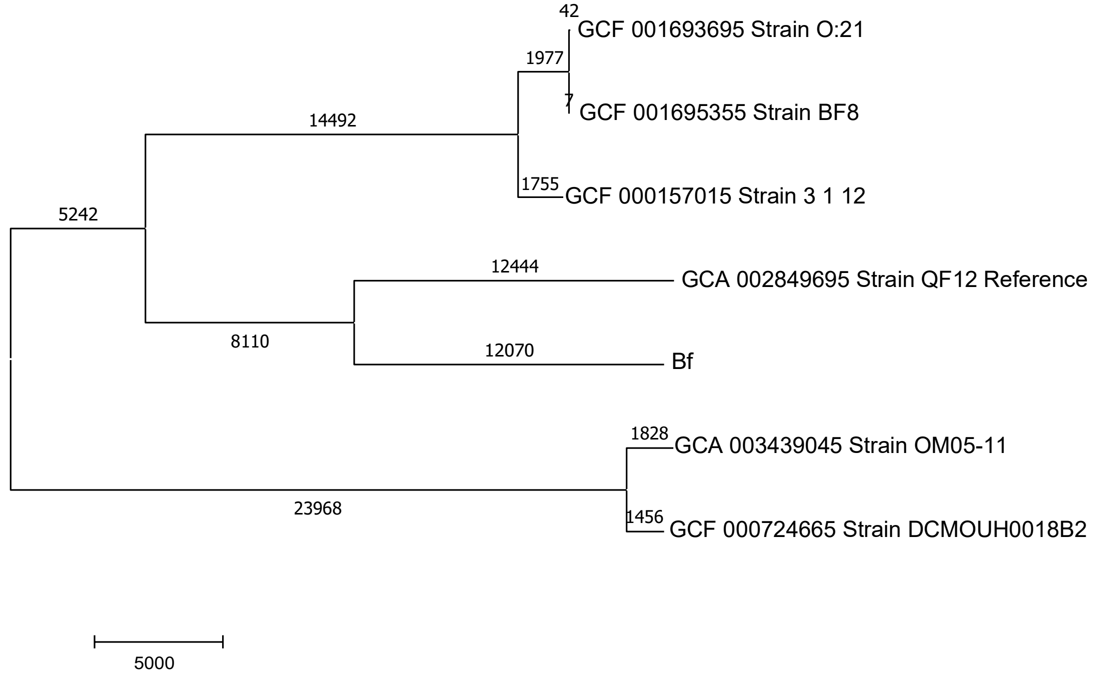
42
GCF 001693695 Strain O:21
1977
7
GCF 001695355 Strain BF8
14492
1755
GCF 000157015 Strain 3 1 12
5242
12444
GCA 002849695 Strain QF12 Reference
8110
12070
Bf
1828
GCA 003439045 Strain OM05-11
23968
1456
GCF 000724665 Strain DCMOUH0018B2
5000

C.

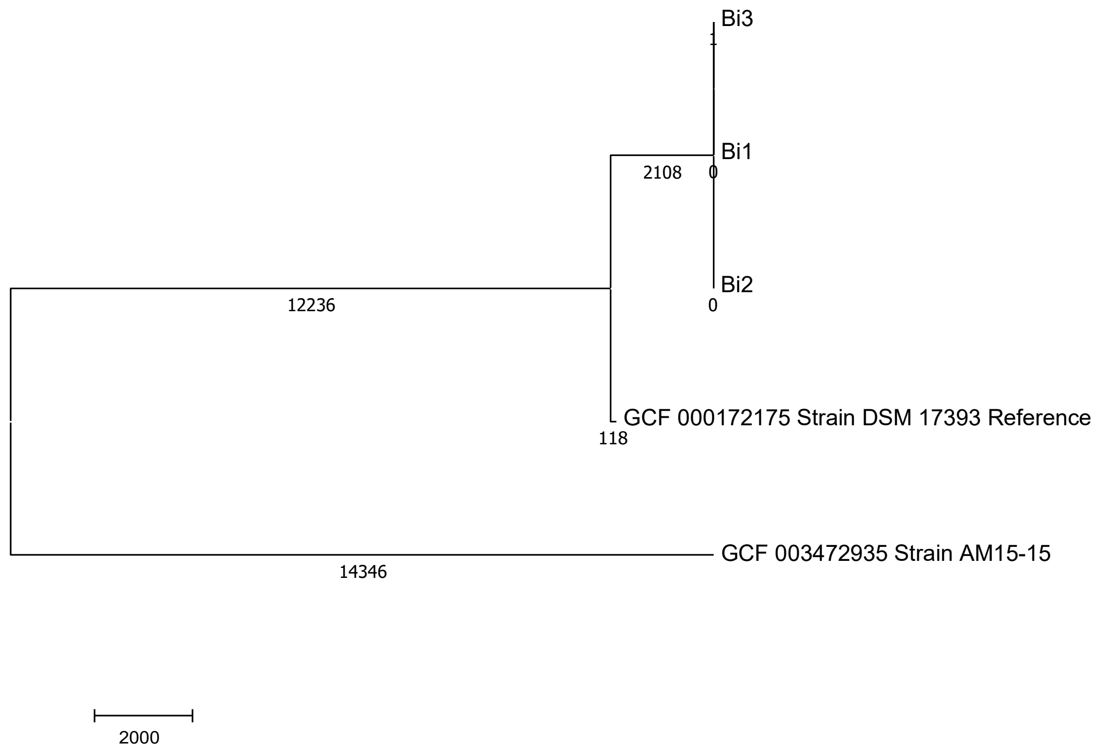
Bi3
1
Bi1
2108
0
Bi2
0
12236
GCF 000172175 Strain DSM 17393 Reference
118
GCF 003472935 Strain AM15-15
14346
2000

D.

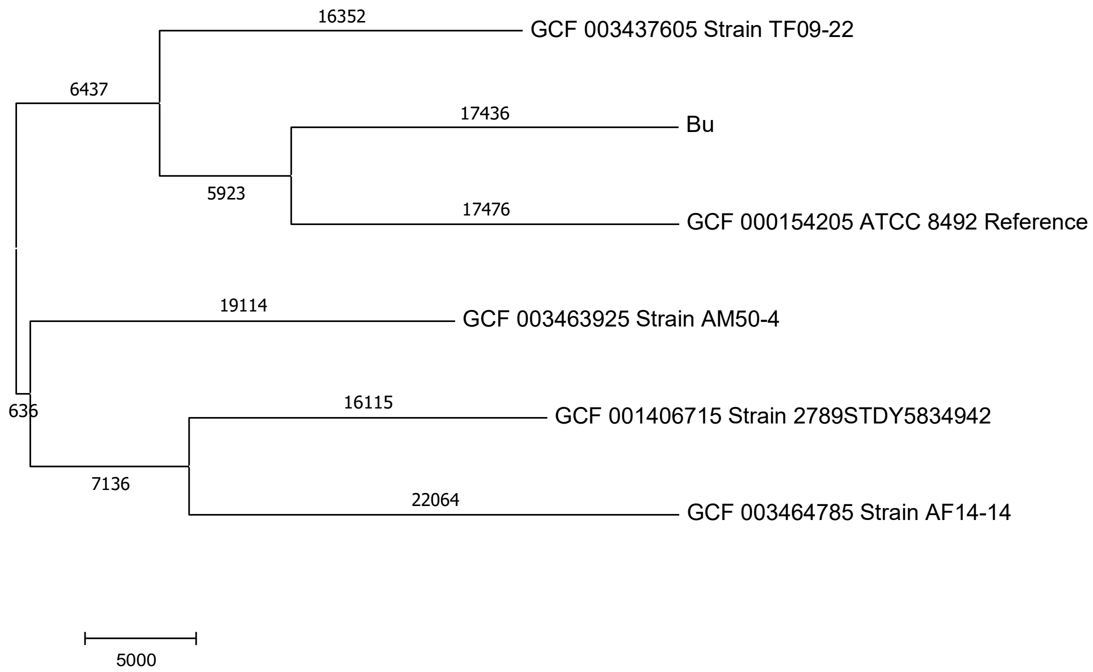
16352
GCF 003437605 Strain TF09-22
6437
17436
Bu
5923
17476
GCF 000154205 ATCC 8492 Reference
19114
GCF 003463925 Strain AM50-4
636
16115
GCF 001406715 Strain 2789STDY5834942
7136
22064
GCF 003464785 Strain AF14-14
5000

E.

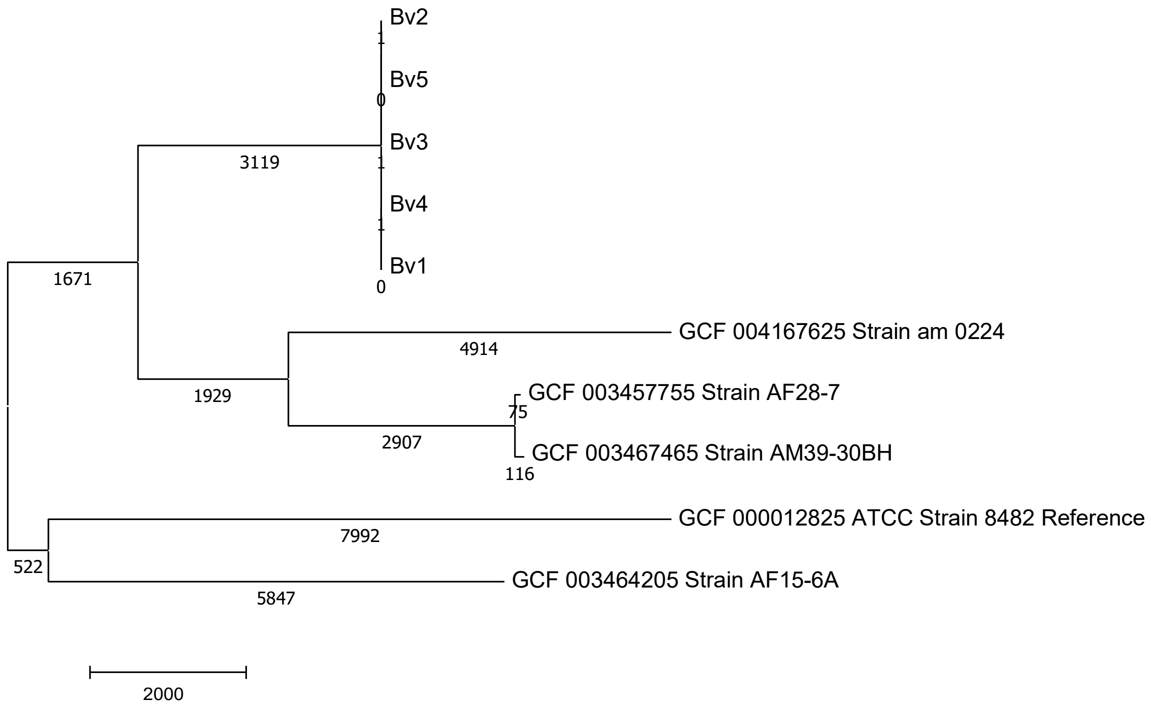


**Figure S1**. Estimates of evolutionary divergence by WGST (number of SNP differences per sequence) between the isolates belonging to the same species and previously published genomes (NCBI database). Bc = *B. caccae* (**A**), Bf = *B. fragilis* (**B**), Bi = *B. intestinalis* (**C**), Bu = *B. uniformis* (**D**), Bv = *B. vulgatus* (**E**). Numbers 1-9 refer to certain isolate. Figures for *P. distasonis* and *B. ovatus* are presented in the text
